# A nanocomposite hydrogel with catalytic properties for trace-element detection in real-world samples

**DOI:** 10.1038/s41598-020-75103-8

**Published:** 2020-10-27

**Authors:** Laura Bertolacci, Paola Valentini, Pier Paolo Pompa

**Affiliations:** 1 Smart Materials, Istituto Italiano Di Tecnologia (IIT), via Morego 30, 16163 Genoa, Italy; 2 Central RNA Laboratory, Center for Human Technologies (CHT), Istituto Italiano Di Tecnologia (IIT), Via Enrico Melen 83, 16152 Genova, Italy; 3 Nanobiointeractions and Nanodiagnostics, Istituto Italiano Di Tecnologia (IIT), via Morego 30, 16163 Genoa, Italy

**Keywords:** Environmental monitoring, Nanoparticles, Colloids, Gels and hydrogels

## Abstract

A nanocomposite material characterized by peroxidase-like properties was developed through the dispersion of platinum nanoparticles (PtNPs) inside a hydrogelic matrix. The integration of the PtNP catalysts within the matrix resulted in their stabilization, preventing aggregation and precipitation in media of environmental interest, characterized by high ionic strength and by the presence of organic solutes. A thorough optimization of the matrix design was aimed at granting optimal diffusion of the reagents, in order to maintain the efficiency of the catalytic action. Such combined features allowed the implementation and prototyping of a colorimetric method for the detection of mercury in environmental water samples. The assay was based on a chromogenic reaction catalyzed by the peroxidase-like activity of PtNPs and its specific inhibition caused by trace amounts of mercury.

## Introduction

The integration of a hydrogelic matrix with metal, non-metal or polymeric nanoparticles (NPs) has been widely employed to formulate nanocomposites—smart materials characterized by a functional combination of the proprieties of the starting components. NPs may confer on nanocomposites specific features such as sensitivity to light, heat or magnetic field (gold, cobalt and nickel nanoparticles), bactericidal potential (gold, silver and copper nanoparticles), catalytic power (platinum nanoparticles), electric conductivity, enhanced mechanical properties etc^1^. In turn, the hydrogelic matrix can provide chemical and physical stability to the material or ease its handling, recover and reuse/recycling. Among the vast spectrum of possible applications of these materials, the main lie in the biomedical field (drug delivery, wound healing, diagnostics), electronics and chemical synthesis (in particular oil cracking and reforming).

A potential unexplored application of these nanocomposite hydrogels regards the development of sensors for the detection of analytes with either environmental or clinical relevance. One of the interesting and widely demonstrated^2,3^ properties of AuNPs and PtNPs is their ability to act as nanoenzymes, mimicking the enzymatic activity of peroxidases, i.e. the catalysis of the redox reaction involving hydrogen peroxide and reducing substrates. Their use in combination with chromogenic substrates, such as 3,3′,5,5′ tetramethylbenzidine (TMB), produces a color change, which can be exploited for sensing purposes by obtaining a naked-eye colorimetric read-out. The general mechanism underlying the use of catalytic nanoparticles for sensing purposes exploits the possibility to quench the color development by rationally inducing NPs surface passivation. Literature counts several methods for the selective passivation of NPs as a direct consequence of the presence or absence of an analyte^4–6^. Their applicability to real samples, when demonstrated, is usually limited to tap or ground water^7–12^. This probably reflects the fact that matrices forming real samples (such as tap water and, even more, environmental and clinical samples) are extremely complex and contain several species responsible for the destabilization of nanoparticles, with consequent loss of catalytic properties. AuNPs and PtNPs owe to their high surface/volume ratio their extraordinary catalytic efficiency, which dramatically overcomes the power of their bulk metal counterparts. On the other hand, this property is granted as long as the system maintains its monodispersity. The labile stability of colloidal suspensions of nanoparticles is a well-known criticality, thus many efforts were spent to develop strategies for the stabilization of nanoparticles^13^. Nevertheless, maintaining the nanoparticles stable and, simultaneously, catalytically active still remains a main issue, especially when dealing with systems characterized by high ionic strength^14^ and presence of organic solutes (proteins or other macromolecules)^15^, such as environmental or clinical samples. These obstacles typically limit the applicability to real samples of the sensing methods based on colloidal nanoparticles.

In this context, we planned to create innovative nanocomposite hydrogels for the development of a sensing strategy in which the catalytic properties of the nanoenzymes confer remarkable sensitivity, while their integration into a solid material successfully overcome the instability issues connected to colloidal suspensions. In particular, to prove the potential of our nanocomposite material, we employed it in a sensing system for the detection of mercury in environmental samples.

The choice of the latter as a model target was motivated by the fact that mercury is a highly toxic element (targeting mainly the central nervous system, the intestine and the kidneys) and, due to its diffusive industrial and medical use, exposure to mercury may be considered universal^16^. Contamination of surface waters causes the entry of mercury in the food chain, with contaminated fish products representing the main source of mercury uptake in the human population^16^. Hence, a strategy for the on-field detection of mercury contamination in environmental water samples would be extremely useful.

## Results and discussion

**Figure 1:**
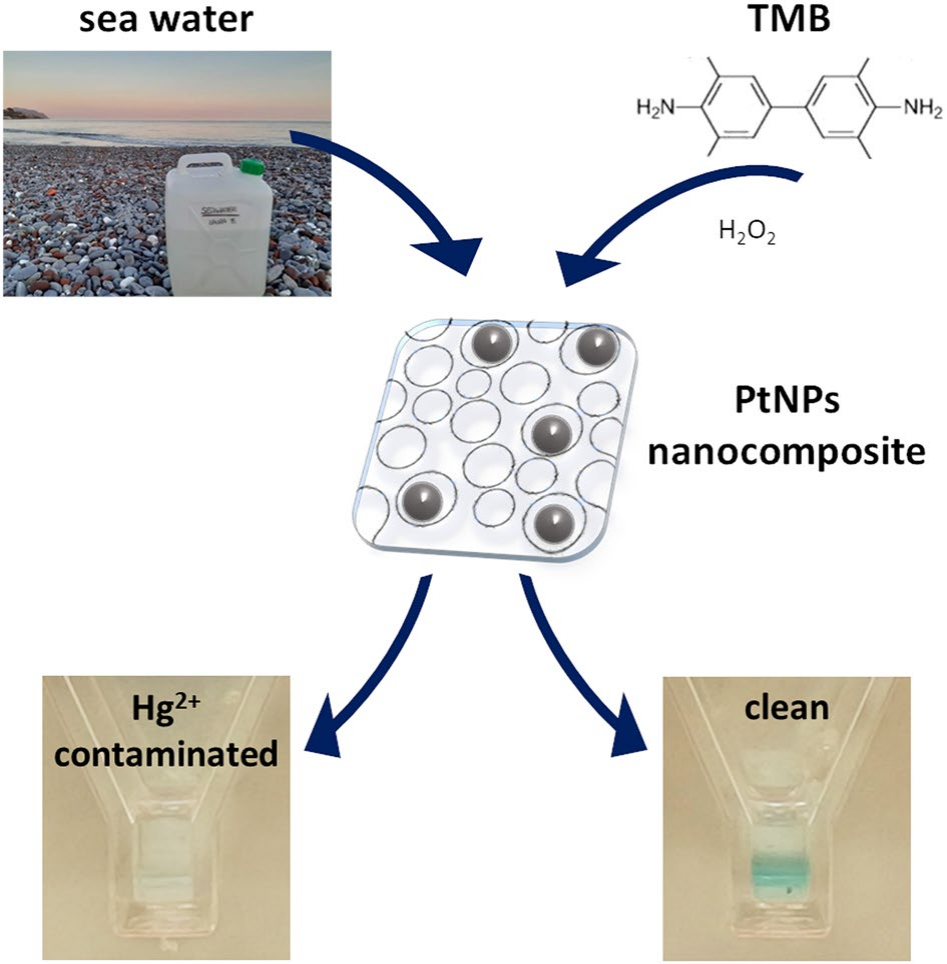
Schematic representation of the principle of detection: embedding PtNPs in the nanocomposites (center) allows their stabilization in the presence of complex environmental matrices (such as sea water) and the preservation of their catalytic properties. Therefore, PtNPs can act as nanozymes to catalyze the chromogenic reaction of TMB and serve as a sensor for Hg^2+^ contamination of water. Indeed, trace amounts of Hg^2+^ inhibit the chromogenic reaction by passivating the surface of PtNPs due to the formation of an amalgam with the latter.

We exploited a known naked-eye detection strategy^8–10^ that is significant for its high sensitivity and selectivity, due to a specific interaction between the mercury/noble metal couple. If present in the sample as a contaminant, cationic mercury is reduced by a weak reducing agent (usually the trisodium citrate present on the surface of the nanoparticles) and forms an amalgam on the surface of the nanoparticles. The consequent passivation of the nanoenzyme surface inhibits its catalytic activity on the chromogenic substrate (TMB). This results in a simple scheme of detection involving two possible states: OFF (no color development) when the analyte is present and passivates the NPs; ON (color development) when the analyte is absent and NPs are not passivated (Fig. 1).

However, as mentioned before, the intrinsic instability of NP colloidal suspensions hinders their use in the presence of complex matrices^17^. Scope of this work was to address this issue through the design of a material combining the catalytic properties of PtNP suspensions with the stability of a solid matrix. The nanocomposite we developed was formed by PtNPs dispersed in a hydrogel. Crucial for our goal was the definition of the optimal porosity of the matrix able to impede NP migration and/or aggregation, while allowing free and fast diffusion of the chemicals employed in the chromogenic reaction catalyzed by the NPs.

### Optimization of hydrogel porosity

The minimal porosity adequate to maintain the NPs dispersed inside the material was experimentally investigated. In a first step, AuNPs were employed to easily screen the hydrogel concentration. Indeed, while PtNPs suspension is transparent at the concentration used in our nanocomposite, AuNPs colloidal suspension is characterized by a red color due to the SPR peak in the visible region (520 nm). This allowed a straightforward monitoring of the presence and stability of the NPs, even at the very low used concentrations, thanks to the high molar extinction coefficient (in the order of magnitude of 10^8^ L mol^−1^ cm^−1^) of the AuNP suspensions at 520 nm. In details, the AuNPs based nanocomposite assumed the expected pale red color, confirming that the NPs remained monodispersed upon their integration within the matrix. Any change in the color of the nanocomposite, upon immersion in test matrices, could be easily correlated to their dispersion outside the material. In a second step, the PtNPs based nanocomposite was developed according with the hydrogelic concentrations previously optimized. Their presence and stability was indirectly confirmed by testing their catalytic properties.

**Figure 2:**
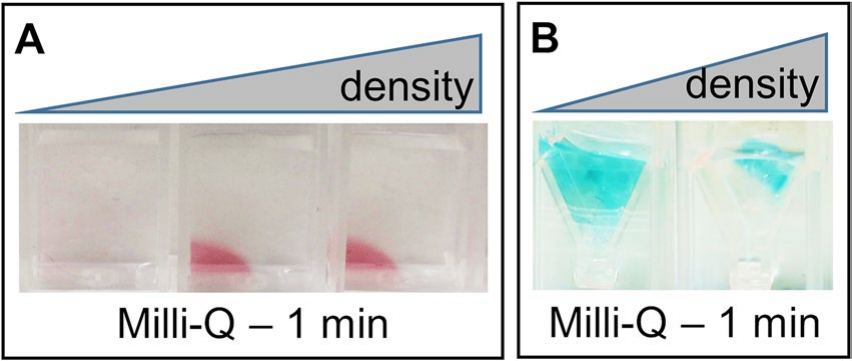
Optimization of hydrogel density. (**A**) Nanocomposite material containing AuNPs, with different density of the polymeric component. When the nanocomposite material is immersed in MilliQ water, NPs tend to diffuse in the surrounding medium and the whole sample becomes pale pink (sample on the left, 0.125%), unless the hydrogel in which NPs are embedded is dense enough to retain them (second and third samples—0.25% and 0.5% respectively—the material remains pink). (**B**) Nanocomposite material containing PtNPs, with different density of the polymeric component. When immersed in MilliQ water, dense hydrogel can retain the NPs (sample on the right, 0.5%) and the catalytic reaction occurs only inside the material, where the nanocatalysts are trapped. If, instead, NPs are embedded in less concentrated hydrogel, they diffuse in the surrounding medium and the catalytic reaction occurs in the whole sample (sample on the left, 0.125%).

An initial screening of hydrogel concentration was performed by immersing the nanocomposites in Milli-Q water. Since AuNP colloidal suspension is red, it was easy to identify by naked-eye the presence of monodisperse NPs in the material, confirming that the hydrogel was dense enough to retain them (Fig. 2A). If instead, the density of the material was too low to keep the NPs trapped inside, they diffused outside; as a result, the color of the material faded, while the surrounding solution assumed a pale pink shade (Fig. 2A). Similarly, the presence of PtNPs in the material was indirectly identified by performing the chromogenic reaction catalyzed by PtNPs. When the NPs were trapped in the material, the chromogenic reaction occurred only inside the latter, which selectively turned blue (Fig. 2B). On the other hand, if the NPs were able to freely diffuse outside the material, the reaction occurred in the whole solution, where the color was homogeneously generated (Fig. 2B, left sample).

**Figure 3:**
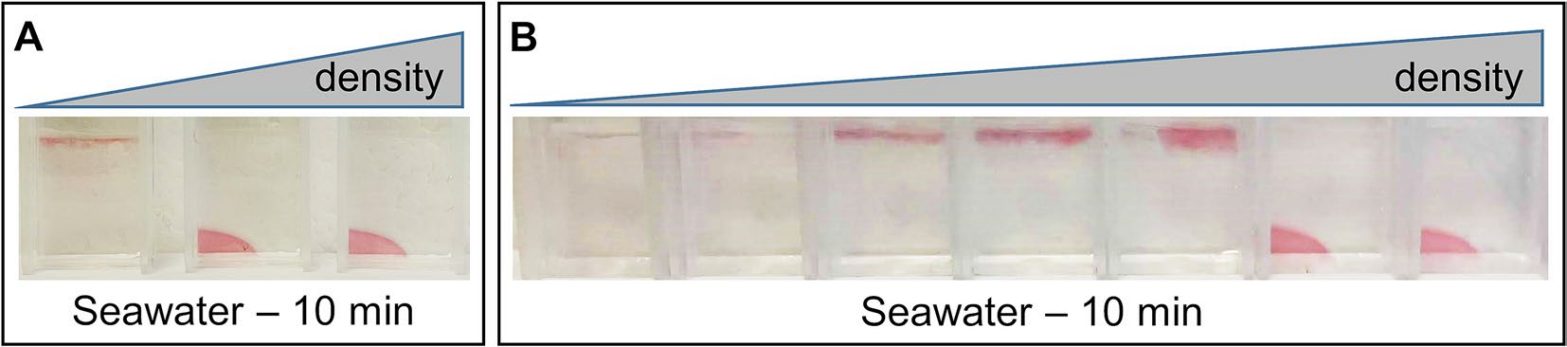
Effects of destabilizing matrices. (**A**) Nanocomposite material containing AuNPs, with different density of the polymeric component. When the nanocomposite material is immersed in seawater, NPs immediately aggregate leading to the disruption of the nanocomposite material (sample on the left—0.125%—some small debris float on the surface), unless the hydrogel in which NPs are embedded is dense enough to avoid aggregation (second and third samples—0.25% and 0.5%—the material remains pink). B) A more detailed screening of concentrations, in the same range of Fig. 2A (0.125%, 0.15%, 0.175%, 0.2%, 0.225%, 0.25%, 0.5%), allows appreciating the dependence of the stabilizing effect of the hydrogel on the density of the material.

Determination of the stability in SW samples of AuNPs-based nanocomposite was straightforward, as aggregation and precipitation of AuNPs can be visually assessed by the loss of the red color. We therefore investigated the correlation between the density of the hydrogel and its stabilizing power in SW. Figure 3 shows the same hydrogels of Fig. 2, immersed in SW instead of MilliQ water. The concentrations that avoided NP diffusion in MilliQ water were also efficient in maintaining NPs stable even in SW (Fig. 3A). At the same time, if the hydrogel was not dense enough to retain the NPs (sample on the left, Fig. 2A), NPs immediately aggregated upon immersion of the nanocomposite in SW, leading to its disruption (sample on the left, Fig. 3A). Figure 3B shows a more detailed screening of hydrogel density, within the same range of concentration of Figs. 2A and 3A. The image clearly demonstrates how the stabilizing effect of the hydrogel correlates with its density.

**Figure 4:**
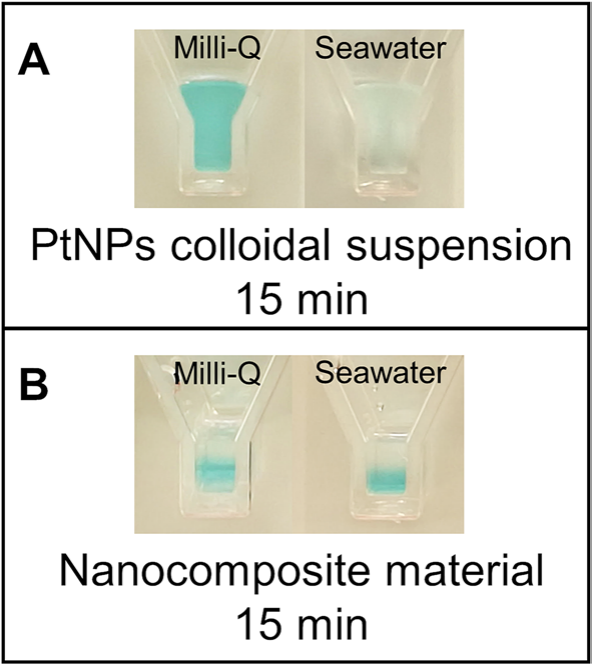
Effect of seawater on PtNPs. (**A**) A colloidal suspension of PtNPs, stable and active when in MilliQ water (the sample turns blue—left), precipitates in presence of seawater and cannot catalyze the chromogenic reaction of TMB oxidation (the sample remains colorless—right). (**B**) On the other hand, if PtNPs are embedded in the hydrogelic matrix, they remain stable and active in both MilliQ and seawater, regardless of the ionic strength.

The optimized characteristics obtained for the AuNP based nanocomposite were used to direct the development of the PtNPs based nanocomposite. To confirm the protective effect of the nanocomposite structure on the catalytic efficiency of PtNPs, we performed the chromogenic reaction catalyzed by PtNPs, either free or embedded in the nanocomposite. The assay was conducted in parallel both in MilliQ water and in SW (Fig. 4A). When working with free PtNPs, the reaction in MilliQ water led to the generation of the expected blue color, while the same reaction in SW had a very weak efficiency (sample on the right). Conversely, in the PtNP-hydrogel system, the reaction occurred with comparable efficiency both in MilliQ water and in SW, as shown in Fig. 4B. This clearly demonstrates that the PtNPs in the SW sample in Fig. 4A precipitated because of the high ionic strength, while the hydrogelic component protected the PtNPs from the precipitation induced by the SW.

### Nanocomposite characterization

**Figure 5:**
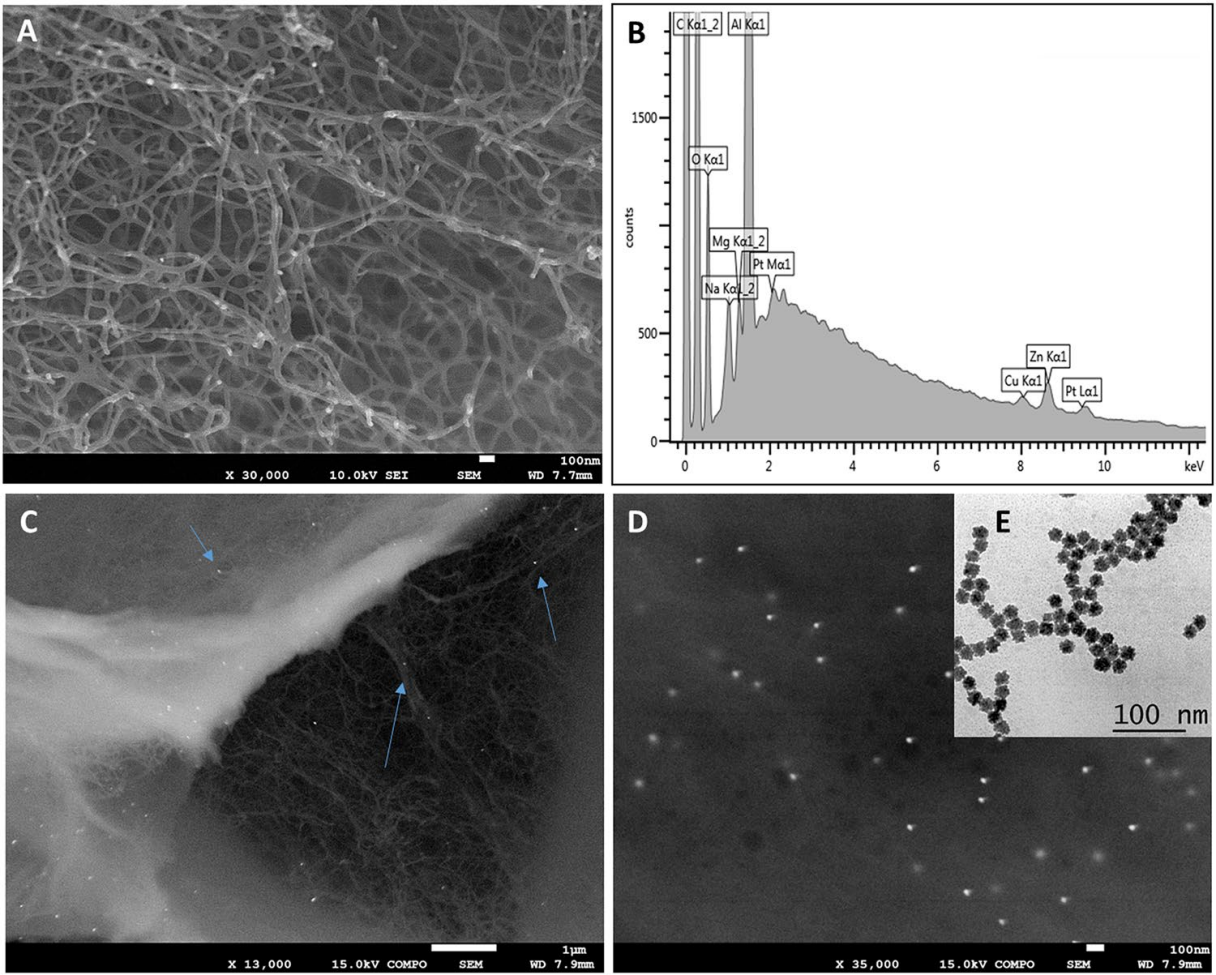
Nanocomposite morphology characterization. (**A**) SEM micrograph of the sample showing the morphology of the nanocomposite and the relative EDS spectrum (**B**) showing the X-ray lines of Pt (Lα1 line = 9441 KeV and M line at 2048 KeV). Backscattered electron micrographs showing (**C**) the morphology of the sample and PtNPs distribution inside the hydrogelic matrix (arrows) and (**D**) PtNPs distribution investigated at higher magnification. TEM micrograph of the synthetized PtNPs confirms their shape and size (**E**).

The morphology of the nanocomposite was investigated by Electron Microscopy. In details, Scanning Electron Microscopy (SEM) allowed the deep investigation of the hydrogelic matrix structure (Fig. 5A). The presence of the platinum nanoparticles inside the matrix was verified by SEM analysis using back scattering imaging mode (Fig. 5C,D). The PtNPs are uniformly distributed within the matrix (arrows) and the presence of Pt peak showed in the EDS spectrum (Fig. 5B) confirms the effective incorporation of the nanoparticles. Transmission Electron Microscopy (TEM) image (Fig. 5E) confirms the shape and size of the nanoparticles. The EDS spectrum shows also the Mg, Na peaks attributable to the hydrogel matrix (Al peak is due to the aluminum stub used to fix the sample for the EDS analysis and the Zn and Cu peaks are attributable to contamination during the dehydration process).

### Mercury detection using PtNPs colloidal suspension

Despite exploiting a known mechanism^8–10^, our passivation strategy differs from the published ones for two main reasons, as previously disclosed in our patent application^18^. In details, during the incubation of the nanozyme with the sample, a buffer at acidic pH and a weak reducing agent are added. The former allows to increment sensitivity, by maximizing the availability of mercury. The latter helps amplifying the effect of NP passivation on the naked-eye readout.

**Figure 6:**
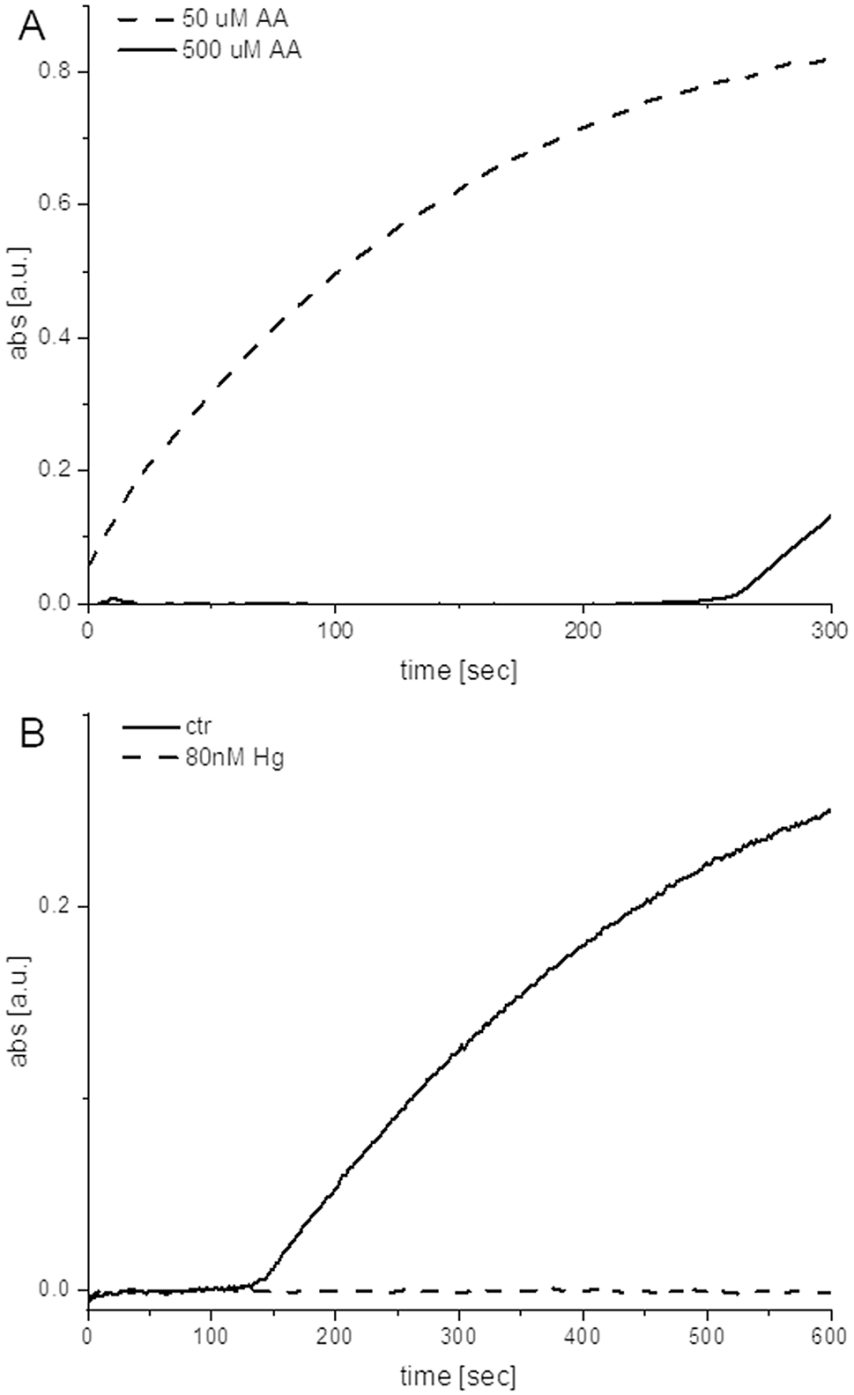
UV–vis spectrophotometric monitoring of the chromogenic reaction. (**A**) Dependence of the signal delay from [AA]: comparison between 50 μM and 500 μM AA. (**B**) PtNPs inhibition: comparison between negative control (ctr—full line) and inhibition due to 80 nM Hg^2+^ (dashed line).

As described in literature^19^, the presence of solubilized salts in the real samples is responsible for the speciation of Hg^2+^ into complex species hardly available for reduction with weak reducing agents. A pre-treatment of the samples with an acidic buffer (10 mM sodium citrate/citric acid pH 5.0) shifts the speciation equilibria towards the free cation, which in turn can undergo the successive reduction. The addition of a weak reducing agent (ascorbic acid—AA) has a twofold effect: as expected, it helps reduction of the mercury cation; moreover, it is responsible for a concentration-dependent delay in the starting of the colorimetric reaction (Fig. 6A). In case of high AA concentrations, the signal of absorbance at 652 nm remains null for a few minutes before starting to grow according to normal absorbance vs time curves . This was justified considering that ascorbic acid competes with TMB for the consumption of H_2_O_2_. As empirically detected, hydrogen peroxide at the beginning oxidizes almost selectively AA; after part or the totality of the latter is consumed, the oxidation of TMB to its coloured metastable intermediate starts, following the traditional behaviour of the redox. This fact is determinant in enhancing the gap between negative and positive readout. In fact, when the PtNPs are passivated by the analyte, they are much less efficient in catalysing the consumption of AA, extending the time delay in the positive samples, before the start of any potential oxidation of TMB and colour generation. This optimized system could easily detect 80 nM Hg^2+^ in tap water, regardless the speciation of Hg^2+^ due to the presence of solubilized salts (Fig. 6B). A thorough analysis of the sensitivity and selectivity of the assay is reported in the Supplementary Information, Figures S1–S5, respectively.

### Mercury detection in real samples

In the case of the analysis of simple samples with limited ionic strength, such as bottled or tap water, the main improvement deriving from embedding PtNPs in a polymeric matrix is that the sample can be removed and the detection mix is added to the nanocomposite during the second step, preventing additional dilution of the nano-particles. Further, this heterogeneous reaction scheme allows the sequential incubation with several aliquots of sample, resulting in preconcentration of the analyte on the NP surface.

Conversely, when dealing with samples from environmental or clinical origin, the use of the nanocomposite material results crucial because, as described before (Fig. 4), the colloidal suspensions of NPs may be not stable in such samples, whereas they remain stable if embedded in the hydrogelic matrix.

**Figure 7:**
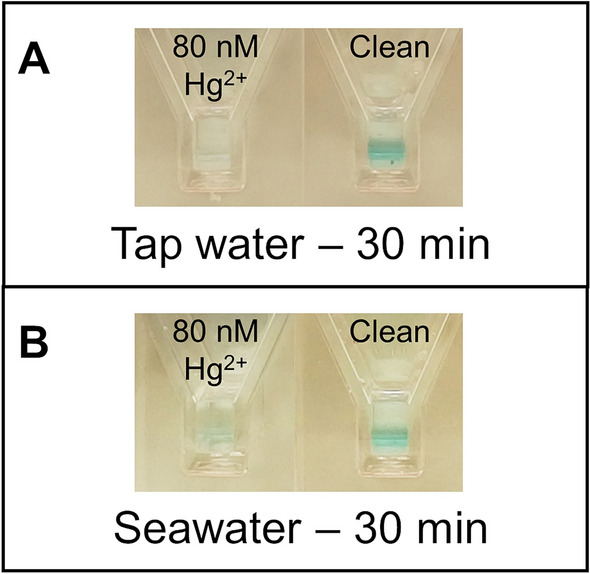
Application of the nanocomposite material for the detection of mercuric ion. (**A**) Tap water. (**B**) Seawater. On the left of each panel, the sample spiked with 80 nM of Hg^2+^, on the right, the negative control.

Figure 7 shows how the nanocomposite material can be employed to detect mercuric ions indifferently in tap water (A) and seawater (B). In a short interval of time (around 15–20 min) the nanocomposite material turns light blue if the sample is not contaminated, while it remains colorless if mercuric ions are present in the sample at concentrations above the law limits. The longer reaction time compared to free NPs was attributed to the slowing effect of the hydrogelic matrix on the diffusion of the reagents.

## Conclusions

The nanocomposite material developed in this study can be described as a colloidal dispersion of nanoparticles, where the dispersing substance is a stable and manageable polymeric solid. This permits to maintain all of the properties of the colloid dispersed within the hydrogelic meshes, including its catalytic activity as a nanozyme, while bearing the advantages of solidity and stability deriving from the surrounding material. An analytical method for mercury detection was developed as an example of applicability of the nanocomposite material in the sensing field. The innovation of our approach resides in the fact that the sensing occurs directly within the nanocomposite material, which can be employed in reaction environments that are prohibitive for NPs colloidal suspensions (such as seawater).

As a further development, our nanocomposite material could substitute colloidal suspensions in a wide class of sensing methodologies relying on the selective modulation of NPs catalysis. Particularly promising are those strategies that apply aptamers^4,5^, oligonucleotides able to affect the nanozyme activity depending on the presence of a specific analyte. Considering the reduced sizes of the aptamers, generally not above 5 nm^20^, it can be reasonably supposed that they are able to easily diffuse through the pores of the hydrogelic material and reach the nanoparticles. Such methodologies, currently, can be applied only to a restricted selection of samples, such as MilliQ water (no analytical utility) or tap water (limited analytical utility). On the other hand, their integration with our nanomaterial would allow extending the use of these methods to real environmental and biological samples, exponentially increasing their analytical relevance.

## Methods

### Platinum NP synthesis and catalytic properties

Citrate-capped NPs with diameter 20 nm (Pt20) were synthesized by the seed-growth method as reported by Moglianetti et al^2^. Briefly 3 mL of seeds, prepared following the procedure reported by Bigall et al*.*^21^, were added to 87 mL of MilliQ water together with 108 μL of 0.5 M H_2_PtCl_6_ (BioXtra grade, Sigma-Aldrich) and 1.5 mL of a solution containing 1.25% (m/v) sodium citrate and 1% (m/v) L-ascorbic acid. The temperature was then slowly raised (~ 5 °C/min) to the boiling point and the reaction was conducted at reflux (~ 100 °C) under stirring for 1 h. The suspension was finally cooled at room temperature. Pt20 were washed with 2 mM citrate solution in 50 K Amicon filters to ensure the removal of any contaminant.

The stability of the Pt20 in different samples (MilliQ water, tap water and seawater—SW) was assessed through a standardized protocol by testing their ability to catalyze the redox between hydrogen peroxide and the chromogenic substrate TMB. In details, 1 μl of pre-diluted stock of PtNPs (125 pM) was mixed with 99 μl of sample (milliQ water or SW) and incubated for 5 min. The sample was then diluted 1:10 in the reaction mix composed by 10 mM sodium acetate buffer (CH_3_COOH/CH_3_COONa) pH 4.5, 200 mM hydrogen peroxide and TMB Substrate Reagent Set RUO (BD OptEIA ) final dilution 1:10. The development of blue color due to the TMB oxidation was detected either by naked-eye inspection, or by UV–vis spectrophotometry. After the synthesis, the size and shape of the Pt nanoparticles were investigated with TEM.

### Synthesis of the nanocomposite materials and optimization of hydrogel porosity

500 pM of either AuNPs (20 nm diameter, supplied by BBI solutions) or Pt20 (prepared as described in the previous paragraph) were dispersed in a water-based solution of the polymer at increasing concentrations, and 50 μl of suspension were deposed on the bottom of a plastic cuvette and let solidify for 30 min. Two simple protocols were developed in order to experimentally identify the minimal porosity adequate to maintain the NPs dispersed inside the material.

### AuNPs based nanocomposite

To empirically determine the optimal concentration of the hydrogel able to stabilize the AuNPs, the material, after solidification, was soaked in 450 μl of MilliQ water. Visual inspection of the samples allowed the individuation of the best condition to avoid diffusion of AuNPs from the hydrogel (see results).

### PtNPs based nanocomposite

PtNP suspensions have a SPR peak in the UV region, with no absorption bands in the visible range. For this reason, although concentrated samples have a brownish to dark-grey color, at the concentration used in our nanocomposite, the sample resulted transparent and it was impossible to detect either the presence or the diffusion of the NPs. On the other hand, the catalytic properties of Pt20 were exploited to detect their presence within the nanocomposite, by performing the TMB test described above.

### Nanocomposite characterization

PtNPs size and shape were determined by using a JEOL JEM 1011 microscope operating at 100 kV of accelerating voltage, after depositing a drop of PtNPs colloidal suspension on a carbon coated grid and drying under vacuum.

The morphology of the nanocomposite was characterized by Scanning Electron Microscopy (SEM). Before the analysis the sample was rinsed for 10 min in increasing concentrations of ethanol (30, 50, 70, 96 and 100%). After ethanol dehydration the sample was placed in the Critical Point Dryer where was flushed in liquid CO_2_ in three consecutive 30 min series and left for 60 min at 35 °C to evaporate all the CO_2_.

Then, the dehydrated sample was fixed on an aluminum stub and coated with a thin layer of carbon. The cross-section morphology of the sample was investigated using a JEOL JSM-7500FA SEM equipped with a cold FEG, operated at 10 and 15 kV of accelerating voltage. The EDS analyses to test the PtNPs doping inside the hydrogelic matrix were performed using an Oxford X-Max 80 system equipped with an 80 mm^2^ silicon drift detector (SDD).

### Mercury detection using PtNPs colloidal suspension

Mercury detection was optimized using PtNPs because of their higher catalytic efficiency compared to AuNPs. First, the PtNPs were incubated with the sample to induce passivation of the surface in case the sample is contaminated with mercury. In a second step, the detection mix was added, in order to visually discriminate between contaminated and not contaminated sample. In details, 88.2 μl of water sample to be analysed were added with 9.8 μl of 100 mM citric acid/sodium citrate buffer solution pH 5.0, 1 μl of Pt20 (125 pM in 2 mM trisodium citrate) and 1 μl of 5 mM ascorbic acid. After 5 min of incubation, the sample underwent the above described TMB chromogenic reaction. In case of clean sample, the suspension turned light blue in a range of time between 4 and 7 min (instrumentally this could be detected as an increase of the adsorption signal above an empirically set threshold of 0.1 absorbance units). In case of contaminated sample, the suspension remained colourless during the same time interval (instrumentally this could be detected as an adsorption signal remaining below an empirically set threshold of 0.05 units).

### Mercury detection using PtNPs nanocomposite

The protocol described in the previous paragraph was upgraded by the substitution of the colloidal PtNPs suspension with the nanocomposite material. The latter was incubated with the water sample, added with citric acid/sodium citrate buffer and ascorbic acid, as described above. After 5 min, the liquid sample was removed and the nanocomposite was soaked in the detection mix. The colour generation, in case of clean sample, occurred within 15 min.

## Supplementary information

Supplementary Information
