# Author Correction: A nanocomposite hydrogel with catalytic properties for trace-element detection in real-world samples

**DOI:** 10.1038/s41598-021-83230-z

**Published:** 2021-02-08

**Authors:** Laura Bertolacci, Paola Valentini, Pier Paolo Pompa

**Affiliations:** 1 Smart Materials, Istituto Italiano Di Tecnologia (IIT), via Morego 30, 16163 Genoa, Italy; 2 Central RNA Laboratory, Center for Human Technologies (CHT), Istituto Italiano Di Tecnologia (IIT), Via Enrico Melen 83, 16152 Genova, Italy; 3 Nanobiointeractions and Nanodiagnostics, Istituto Italiano Di Tecnologia (IIT), via Morego 30, 16163 Genoa, Italy

Correction to: *Scientific Reports* 10.1038/s41598-020-75103-8, published online 27 October 2020

The original version of this Article contained an error in the Author Information section where:

“These authors contributed equally: Paola Valentini and Pier Paolo Pompa.”

now reads:

“These authors jointly supervised this work: Paola Valentini and Pier Paolo Pompa.”

This error has now been corrected in the PDF and HTML versions of the Article.

