# Supplementary material for: A nanocomposite hydrogel with catalytic properties for trace-element detection in real-world samples
Source: Sci Rep. 2020 Oct 27;10:18340. doi: 10.1038/s41598-020-75103-8 (PMC7591478; doi:10.1038/s41598-020-75103-8)
Supplement: Supplementary file 1 — Supplementary Information [file 41598_2020_75103_MOESM1_ESM.pdf]

A nanocomposite hydrogel with catalytic properties for trace-element detection in real-world samples.

Laura Bertolacci, Paola Valentini, Pier Paolo Pompa.

Assay Sensitivity

Sensitivity was investigated by analysing laboratory tap water spiked with different concentrations of Hg^{2+}: 0 nM , 50 nM, 80 nM and 100 nM.

Besides the evident visual readout, the colour change was monitored spectroscopically in order to acquire and store the absorbance vs time curves of all the samples.
A parameter to discriminate between positive and negative result from the instrumental output was defined. In details, a first threshold to 0,05 a.u. was set, below which the sample remains clearly colourless, equivalently a second threshold of 0,1 a.u. was defined above which the sample turns clearly light blue. In between the two values, since the colouration is a continuous process and the perception of the colour is subjective, the nature of the result cannot be stated reliably.

It is evident from Supporting Figure S1 that the control sample turns blue within few minutes (absorbance reaches the 0,1 a.u. threshold after around six minutes and overcomes 0,2 at the end of the monitoring, after 10 minutes). On the other hand, samples spiked with both 80 nM and 100 nM Hg^{2+} remain colorless throughout the whole experiment (absorbance at the baseline). Finally, the sample spiked with 50 nM Hg^{2+} remains colourless for more than 8 minutes (absorbance below the 0,5 a.u. threshold), while at the end of the experiment, the absorbance lays between the two defined threshold values, where the nature of the result cannot be stated reliably by naked eye, since the perception of the colour is subjective.

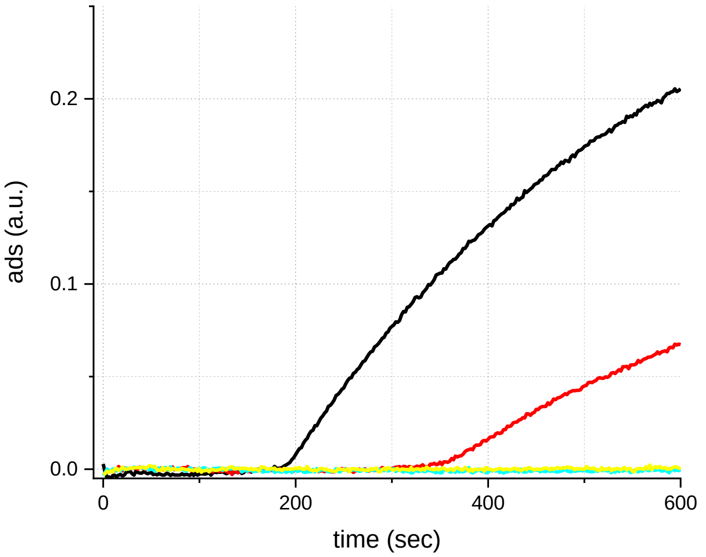

Figure S1. Investigation of the inhibitory effect of increasing Hg^{2+} concentrations on chromogenic reaction catalyzed by PtNPs. Tap water from our laboratory was spiked with 50 nM (red line), 80 nM (yellow line) and 100 nM Hg^{2+} (light blue line). Unspiked sample (black line) was tested as negative control. All the concentrations of cationic mercury lead to a strong inhibition of the catalytic reaction, but the lower concentration (50 nM) gave a result that did not pass our defined acceptance criteria.

For this reason, we decided to confirm 80 nM as LOD of the method (which is amenable of further optimization by acting on the analysis parameters) and to employ it as standard concentration to spike for the validation step in real water samples.

Validation was performed by analysing in duplicate 95 tap water samples from different Italian cities before and after spiking with 80 nM Hg^{2+}. Again, visual readout was coupled with systematic spectroscopic analysis.

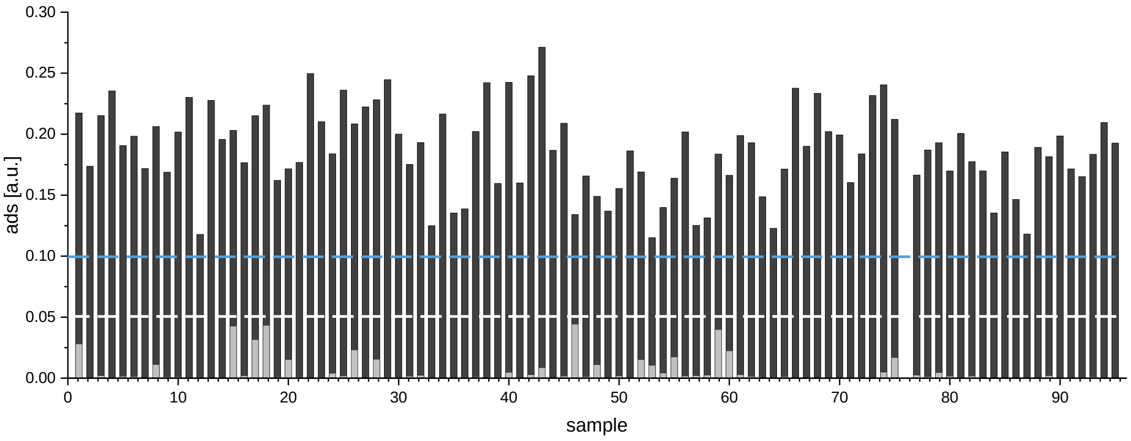

Figure S2. Absorbance of the samples after 7 minutes of analysis. Negative controls are in dark gray, while the absorbance at 7 minutes of the same samples spiked with 80nM Hg2+ is shown superimposed in light grey. For each sample a consistent decrease in the absorbance is induced by the presence of mercuric cations. Blue line indicates the 0.1 a.u. absorbance threshold above which the samples appear clearly coloured by naked eye; white line indicates the 0.05 a.u. absorbance threshold below which a visual inspection of the samples does not detect any colour. Sample n°76 remained colourless even in absence of mercury. This is due to a high concentration of Ag^{+} used for disinfection porposes.

The analysis of the results (Supporting *Figure 2*) confirms that the solution of 99% of the negative controls turns light blue within seven minutes as confirmed by values of absorbance above 0.1 a.u., only one sample remained colourless (absorbance below 0,5 a.u.). On the other hand, the solution of **all the spiked samples remains colourless within this interval** indeed the measured absorbance does not reach the 0,05 a.u. threshold.

We further anaysed by ICP-OES the control sample which remained colourless and confirmed, as expected, a high concentration of Ag^{+}, 42.1 ppb (390 nM). This concentration of silver is in agreement with the average range of [Ag^{+}] detected in tap waters treated with silver for disinfection purposes, which sets around 50 ppb (465 nM). On the other hand, average silver concentrations in natural waters are 0.2–0.3 µg/litre (below 3nM).

The attempt to reduce the limit of detection at 60nM (Supporting Figure 3) was not fully satisfying, as only 70% of the spiked samples remained colourless after 7 minutes. Nevertheless, all the samples showed a strong decrease in the colour development compared to the corresponding negative controls. Further optimization of the assay setup could lead to a lower naked-eye LOD.

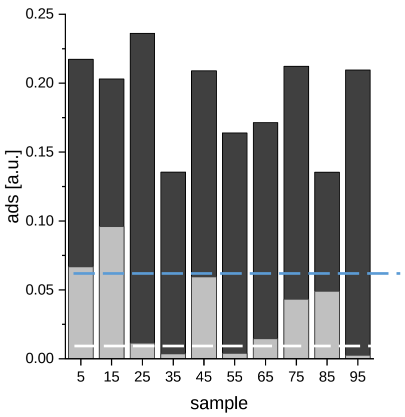

Figure S3. Absorbance of the samples after 7 minutes of analysis. Negative controls are in dark gray, while the absorbance at 7 minutes of the same samples spiked with 60nM Hg2+ is shown superimposed in light grey. For each sample a consistent decrease in the absorbance is induced by the presence of mercuric cations. Blue line indicates the 0.1 a.u. absorbance threshold above which the samples appear clearly coloured by naked eye; white line indicates the 0.05 a.u. absorbance threshold below which a visual inspection of the samples does not detect any colour. 70% of the spiked samples are below the 0.05 a.u. threshould, while 30% lay between 0.05 and 0.1 a.u.; although they didn't developed a clearly blue colour after 7 minutes, they cannot be assumed to be completely colourless.

Assay Selectivity

The mechanism underlying the detection of Hg^{2+} relies on the reduction of the latter to elemental Hg and the formation of an amalgam with the Pt on the surface of the NPs. This gives an outstanding selectivity to the system, thanks to the very high redox potential of Hg^{2+}, responsible of its reduction even in presence of weak reducing species like ascorbic acid (E° 0.39). The latter cannot reduce the majority of cations naturally occurring in fresh waters, such as the lighter alkali metals and alkaline earth metals and the most common transition metals like iron, nickel and manganese, because they all have much lower redox potentials (Supporting Table 1).

Cathode (Reduction) Half-Reaction	*Standard Potential E° (volts)*
$K^+(aq) + e^- \rightarrow K(s)$	-2.92
$Ca^{2+}(aq) + 2e^- \rightarrow Ca(s)$	-2.76
$Na^+(aq) + e^- \rightarrow Na(s)$	-2.71
$Mg^{2+}(aq) + 2e^- \rightarrow Mg(s)$	-2.38
$Mn^{2+} + 2e^- \rightarrow Mn(s)$	-1.19
$Al^{3+}(aq) + 3e^- \rightarrow Al(s)$	-1.66
$Zn^{2+}(aq) + 2e^- \rightarrow Zn(s)$	-0.76
$Cr^{3+}(aq) + 3e^- \rightarrow Cr(s)$	-0.74
$Fe^{2+}(aq) + 2e^- \rightarrow Fe(s)$	-0.41
$Cd^{2+}(aq) + 2e^- \rightarrow Cd(s)$	-0.4
$Co^{2+} + 2e^- \rightarrow Co(s)$	-0.28
$Ni^{2+}(aq) + 2e^- \rightarrow Ni(s)$	-0.23
$Sn^{2+}(aq) + 2e^- \rightarrow Sn(s)$	-0.14
$Pb^{2+}(aq) + 2e^- \rightarrow Pb(s)$	-0.13
$2H^+(aq) + 2e^- \rightarrow H_2(g)$	0
$Cu^{2+}(aq) + 2e^- \rightarrow Cu(s)$	0.34
**dehydroascorbate + $2e^-$ + $2H^+$ $\rightarrow$ ascorbic acid**	**0.39**
$Ag^+(aq) + e^- \rightarrow Ag(s)$	0.80
$Hg^{2+}(aq) + 2e^- \rightarrow Hg(l)$	0.85

Table S1. Standard Redox Potentials (E°) of the main metals occurring as cations in freshwaters. The reduction semi-reaction of dehydroascorbate is shown in light blue. Only cation with higher E° can be reduced to elemental form by ascorbic acid.

Only cation with E° higher than AA (lowest positions in table 1) can be reduced to elemental form by AA, according to the following redox reaction (generalized for a bicharged cation):

$Me^{2+}_{(aq)}$ + ascorbic acid → $Me^{0}_{(\downarrow)}$ + dehydroascorbate + $2H^{+}$

As expected, the chromogenic reaction catalyzed by PtNPs could be performed indistinguishably in MilliQ water and bottled water (*Supporting Figure 4*), confirming that the salts solubilized in drinking water (*Supporting Table 2*) do not affect the assay, despite their relatively high concentration (one to three orders of magnitude higher than the analyte LOD).

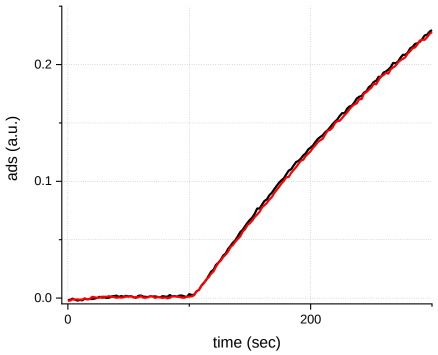

Figure S4. Chromogenic reaction conduced both in MilliQ (red line) and in bottled water. (black line) The signals of the two samples are perfectly superimposable, confirming that salts solubilized in drinking water do not affect PtNPs catalytic activity.

**Bottled water chemical composition as labelled by the manufacturer**					
**Cation**	**[mg/L]**	**µM**	**Cation**	**[mg/L]**	**µM**
Calcium	32	300	Sodium	2.10	20
Magnesium	5.50	50	Potassium	0.65	5

Table S2. Mineral composition of the bottled water tested (cations): Ca^{2+}, Mg^{2+}, Na^{+}, K^{+}.

Elements with E° closest to ascorbic acid (Pb^{2+}, Cu^{2+} and Ag^{+} – E° -0.13, 0.34 and 0.80 V respectively) were tested to evaluate any potential interference. Pb^{2+} and Cu^{2+} did not affect the system at a concentration 10 times higher than that of Hg^{2+}. *Supporting Figure 5* represents the absorbance after 5 minutes: while 100nM Hg^{2+} completely quenches the color development, 1µM Pb^{2+} has no effect and Cu^{2+} has some inhibitory effect that can only be appreciated by instrumental analysis and not by naked eye, indeed the absorbance reaches values much above the 0.1 a.u. threshold. On the other hand, silver can give false positive results, when present at concentrations in the order of magnitude (or higher) of our LOD for Hg^{2+}, due to the similar E° of the two metals. For instance, during the validation test on the tap waters collected from different Italian sites, one sample could inhibit the PtNPs catalytic activity because of the presence of 390nM Ag^{+} (about 40 ppb). It must be highlighted that this concentration derives from the intentional treatment with silver for disinfection purposes, thus can be present only in tap water that underwent this antimicrobial process. On the opposite, average silver concentrations in natural waters are 0.2–0.3 µg/litre (below 3nM) and do not inhibit PtNPs catalytic activity (tested at 10nM, Supporting Figure 5).

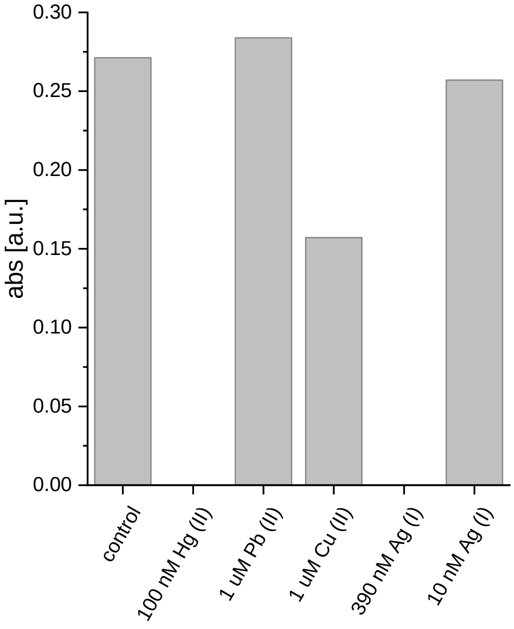

Figure S5. Selectivity analysis. PtNPs can catalyze the color change even in presence of Pb or Cu at a concentration one order of magnitude higher than our Hg^{2+} LOD. Ag^{+}, the only metal with E° very close to mercury one, could inhibit the reaction when present at high concentrations (400nM). Silver can be present at this concentration when used for tap water disinfection. On the other hand, average silver concentrations in natural waters are 0.2–0.3 µg/litre (below 3nM) and tested 10nM do not inhibit PtNPs catalytic activity.
